# Diverse Linguistic Development in Prelingually Deaf Children with Cochlear Implants

**DOI:** 10.1155/2019/1630718

**Published:** 2019-11-21

**Authors:** Pia De Stefano, Francesco Pisani, Giuseppe Cossu

**Affiliations:** ^1^Neurology Unit, Department of Clinical Neurosciences, Geneva University Hospitals, Geneva, Switzerland; ^2^Child Neuropsychiatry Unit, Medicine and Surgery Department, University of Parma, Italy; ^3^Medical Centre of Phoniatrics, Padova, Italy

## Abstract

The advent of cochlear implants has enormously improved the quality of sensory perception in deaf children. Notwithstanding these advantages, the current literature shows a substantial variability in language proficiency among implanted children. This case series explores the variability of language acquisition in congenitally deaf children with cochlear implants. We report 4 prelingually deaf children (mean age = 10.5; SD = 1.08), affected by a genetically determined bilateral deafness, due to GJB2 gene mutation Cx26. Each implanted child underwent a systematic assessment of speech perception and production, as well as of lexical, morphologic, and syntactic skills in both comprehension and production. Notwithstanding similar clinical histories and similarly good postimplant pure-tone audiometry, two of the four children fared very poorly in speech audiometry, whereas the other two children gained very good results. We suggest that the language impairment detected in (some) implanted children may not be fully accounted for by pure auditory thresholds and that may be the outcome of concomitant damage to core components of the child's linguistic brain.

## 1. Introduction

Before the advent of cochlear implants (CI), most children with profound prelingually hearing loss were reported to lag in language acquisition with respect to their hearing peers [1–3]. The beneficial effects of hearing aids (HA) on language development were burdened by limitations in the lexical-semantic, syntactic, and morphological domains [4–6]. Furthermore, quite often, the degree of language impairment persisted over time, in spite of a systematic and prolonged remedial training [7]. Subsequent linguistic analyses confirmed that most children with a profound congenital deafness developed language abilities at approximately half the rate of their peers with normal hearing [5] and presented with long-lasting difficulties in the domain of literacy as well [8]. The linguistic fate of the congenitally deaf children was deeply changed by the advent of CI [9, 10].

Implanted children appeared to acquire language more rapidly and efficiently than those congenitally deaf children who benefited from HA [5, 11]. Furthermore, the acquisition of language and reading following CI was reported to unfold at a rate comparable to that observed in hearing children with similar initial language skills [12, 13].

It is widely acknowledged that the positive effects of CI on language acquisition are induced by the improvements in the fine-tuning of auditory perception, although other variables, such as the age of implant, are claimed to play a role [14, 15].

Nevertheless, the variability in the language performance of CI children remains quite high [16] especially at interindividual level [17, 18]. A longitudinal study [19] corroborates these findings by showing that 10 out of 22 implanted children acquired grammatical proficiency at pace with normal-hearing children, whereas 12 children lagged well behind. Even more recent studies suggest that children with CI may have trouble with language skills and phonological awareness [20–22].

The aim of our study was to investigate the linguistic system in a homogenous group of 4 CI children, affected by a genetically determined bilateral deafness, due to GJB2 gene mutation Cx26, with similar clinical histories and similarly good postimplant pure-tone audiometry, in order to understand if linguistic deficits in these implanted children were secondary to the auditory problem from birth or could be the result of a primary impairment of the linguistic system and consequently of the brain structures involved in language.

## 2. Materials and Methods

We examined four children (mean age = 10.5; SD = 1.08), affected by a genetically determined bilateral congenital deafness. All children presented with a GJB2 gene mutation that interferes with the proper coding of the gap junction channel protein connexin 26 (Cx26) [23]. They were born from hearing parents and were free from neurological, emotional, or behavioural disorders. Unaided preimplant auditory threshold level was ≤90 dB (PTA) for each one of the four deaf children, whereas the aided hearing threshold level, averaged across 0.25-2 khz, was ≤65 dB. They had been using HA for approximately 3 years, and during that time, each child followed regularly a rehabilitation program with a speech therapist. The four deaf children received a CI between 3.8 and 5.2 years of age, and they have been using their own CI for about 5 years. Postimplant auditory threshold (detected at 0.5-4 khz) improved markedly, and at the time of our testing, it ranged from 25 dB to 37 dB (Table 1).

Furthermore, all four children had a high nonverbal intelligence level; on the WISC-R scale [24], they obtained a nonverbal IQ score ranging from 100 to 115. Notwithstanding these findings, the verbal IQ is markedly below the normal level for Participant 1 and Participant 4, whereas it is within a normal range for the other two children (Table 2).

In order to provide normative scores for the production of flexional and free morphology in narrative language, we recruited a control sample of 15 second graders (9 males and 6 females) from a local elementary school. Owing to a ceiling effect in correct responses, we selected a sample of younger children, whose mean age ranged from 7.09 to 8.10 (mean = 7.47; SD = 1.04). Their cognitive level was assessed by means of the Raven Coloured Matrices (PM) [25]; all the control children scored within the normal boundaries (75 to 95 percentiles) on this test for nonverbal intelligence. Children with known or suspected history of brain or behavioural problems were not included in the control sample.

### 2.1. Measures

All four children with congenital deafness and children of the control sample underwent a systematic assessment of language skills: speech perception and production were assessed by means of speech audiometry, according to standard clinical procedures, requiring the repetition of 20 words, presented at different degrees of loudness. We also investigated the lexical comprehension and production, by means of the Peabody Picture Vocabulary Test (PPVT) [26] and the Boston Naming Test [27, 28], respectively. The Test for the Reception of Grammar (TROG) [29] was adopted for the assessment of the children's morpho-syntactic comprehension. As a further tool for the assessment of syntactic comprehension, we also presented each implanted child with an original Italian Test of Grammatical Comprehension for Children (TCGB) [30]. Normative scores are provided for an age range from 3.6 to 8 years of age. The TCGB comprises 76 sentences, aimed at assessing 8 different grammatical structures (locative, flexional, active affirmative, active negative, passive affirmative, passive negative, relative sentences, and dative sentence) and a figured album. For each sentence produced by the examiner, the child is required to point to the correct picture among four alternatives. The normative scores are calculated on the number of errors.

In order to assess bound and free morphology in sentence production, we presented each child with a set of 10 brightly coloured cartooned pictures of common daily life actions and situations (picture numbers 5, 10, 13, 15, 24, 28, 29, 31, 39, and 48). These pictures were taken from the “Prove per la valutazione fonologica del linguaggio infantile” (PFLI) [31], an Italian test devised to elicit a short narrative production in children. Each picture was presented one at a time, and the child was required to look carefully and to tell a short story by describing what “was happening.” No time limitations were set on the child for the description of the picture. If the child produced very few or short sentences (or she kept silent), the examiner provided the child with neutral prompt, such as “Well, look carefully, what's going on here? Tell me more about it.” The children's “stories” (from both the clinical and the control samples) were tape-recorded and then transcribed by the examiner for scoring and further inspection. We only considered the number of errors in the use of free and bound morphemes (i.e., determiners, prepositions, gender, and number for nouns and verbs).

Furthermore, in order to explore the production of prepositions and of pronominal clitics (in sentences of the kind “Io lo mangio” [I eat it]; “Lei li vede” [She sees them]), we adapted to our purpose an act-out toy game devised for eliciting the production of sentences, as from the Neuropsychological Preschool Test (TNP) [32]. In particular, we selected 30 actions that the child was required to name, after viewing the manipulation of toys acted out by the examiner: 15 sentences required the naming of an action including a preposition, whereas the other 15 sentences elicited the production of pronominal clitics. The order of presentation for the two types of sentences to be elicited was randomly interspersed.

## 3. Data Analysis

For language investigations (Peabody Picture Vocabulary Test, TROG, Boston Naming Test, TCGB, TNP, and PFLI), we calculate for each participant *z* score (compared to normative data and for PFLI compared to control group) and the alpha was set at 0.05.

Data were analyzed using S.P.S.S. Statistics 20 software.

## 4. Results

The language performances of the four deaf children show a remarkable intersubject discrepancy in both degree and quality of language skills. As we have shown in Table 1, postimplant audiometry indicates that all four children achieve an equally efficient level of hearing threshold: their hearing level being stable at 25-38 dB between 250 and 4000 Hz. Inspection of speech perception, on the contrary, reveals that the percentage of correctly repeated words vary to a high degree among the four implanted children: at 50 dB, for instance, Participant 1 and Participant 4 achieve a performance of 40 and 25% correct responses, respectively, whereas Participant 2 and Participant 3 achieve a performance of 90 and 70% correct responses, respectively. These preliminary findings from speech perception reverberate across all of the linguistic variables that we have examined. From Table 3, for example, we can observe that both lexical comprehension and lexical production are plainly efficient in Participant 2 and Participant 3, whereas for Participant 1 and Participant 4, the lexical competences are clearly below the normal range.

In particular, for Participant and Participant 4, a comparison with the normative data for the Boston Naming Test [27, 28] yields a *z* score of 4.43 (*p* < .0001) and 2.75 (*p* < .0030), respectively; for Cases 2 and 3, on the contrary, we obtain a *z* score of 1.45 (*p* < .073) and 0.06 (*p* < .476), respectively. Similarly, the responses provided on the Lexical Comprehension Test (PPVT) [26] by the implanted children are within normal range for Participants 2 and 3, whose raw scores correspond to 12.3 and 16 years of age equivalent, respectively. Raw scores from Participant 1 and Participant 4, on the contrary, are significantly below the normal range, as they correspond to 5.3 and 4.9 years of age equivalent, respectively.

The assessment of the comprehension of grammar (by means of the TROG) [29] brings to light a marked intersubject discrepancy between the linguistically “efficient” and the linguistically “inefficient” implanted children. As evident from Table 4, for Participant 1 and Participant 4, all the language scores considered by the TROG are significantly below the normal range.

Quite the opposite for Participant 2 and Participant 3, whose scores are, in some instance, even higher than the control's, the discrepancy from the normative scores is particularly telling on the grammatical score section for Participant 1 (*z* = 7.97, *p* < .0001) and Case 2 (*z* = 6.235, *p* < .0001), respectively. No significant differences are detectable between the other two participants and the normative data.

Similar findings emerge from the Test of Grammatical Comprehension (TCGB) [30], which provides normative data up to the age of 8 years for flectional and free morphology, besides syntactic structures (such as passive negatives and object relative) for the Italian language. Notwithstanding the chronological difference between the (younger) normative sample and the implanted children, the results (Table 5) from Participant 1 and Participant 4 show that the mastery of some important morphological and syntactic structure is beyond the reach of the two linguistically impaired implanted children, whereas the other two children make no error, as expected from children of their age and intelligence.

We also elicited the production of short single-picture stories (PFLI), aimed at monitoring the accurateness of free and bound morphemes in the children's narrative language. In Table 6, we outline the number of morphological errors made by the implanted children and by the (younger) control sample.

In the test eliciting the description of pictures, Participant 1 and Participant 4 either omit or fail to select the correct determiners and prepositions 19 and 32 times, respectively, whilst Participant 2 and Participant 4 total 1 omission. Furthermore, Participant 1 and Participant 2 make a total of 17 errors in the selections of the proper agreement for number and gender with nouns, adjectives, and verbs; the other two children make no error. When presented with picture no. 5 (representing one boy skiing and one girl on a sledge in a snowy mountain), Participant 1 describes the picture in the following way: “... e ci sono ^∗^le slitta ^∗^sciare ... tutti e due sempre ^∗^vuole andare ^∗^montagna perchè ^∗^tanti neve” (...and there are ^∗^the [fem plur - violates gender agreement] sledge [fem. sing] ^∗^ski [inflected verb form -missing preposition] ...both always ^∗^wants [violating number agreement] to go ^∗^mountain [missing the preposition] because many [plur masc – violates gender and number] snow [fem sing]”. The description of the same picture provided by Participant 2 is the following: “..ci sono due bambini che stanno in montagna; un bambino sta sugli sci e invece la bambina sta sulla slitta. Giocano con la neve” (“There are two children who stay up in the mountain; one boy is staying on the skis and the girl, on the contrary, is staying on the sledge. They play with the snow”).

When presented with picture no. 3 (reproducing an old man holding a wrench and watching a sink with the water overflowing on the floor), Participant 3 says: “E' un idraulico che sta aggiustando un rubinetto che perde; però c'è tanta acqua per terra, ma l'idraulico ripara il tubo” (“He is a plumber, who is repairing a tap that leaks; however, there is a lot of water on the floor, but the plumber repairs the pipe”). Participant 4 describes the same picture in the following way “^∗^Ha uscita l'acqua, ... allora è ^∗^caduto l'acqua. Lui ^∗^mette a posto ... qui un puntino rotto nel bagno poi lui chiama la persona. Tanto lui non ^∗^sa fare ... lui chiama una persona per fare un'altra ^∗^ ...no!” (“^∗^Has [incorrect auxiliary verb –for “is”] gone out the 14 water... then is ^∗^fallen [violates gender agreement] the water. He ^∗^sets up [missing direct object] here a little spot broken in the bathroom then he calls the person. Well he is unable ^∗^to do [missing the clitic – it-]... he calls a person to do another ^∗^[missing the direct object]... no!.”). Similarly, clear differences among the four children emerge in the sentence production tasks that require the naming of prepositions and pronominal clitics. In the preposition task, Participants 1 and 4 score 5/15 and 6/15 correct responses, respectively, whereas the other two children gain a correct score of 13/15 and 14/15 each. In the other syntactic test, eliciting the production of clitics, Participant 1 and Participant 4 produce no correct sentence, by omitting all of the required 15 pronominal clitics, whereas Participants 2 and 3 total 8/15 and 13/15 correct responses, respectively.

## 5. Discussion

The results of the present study show that the acquisition of language in some congenitally deaf children may follow discrepant trajectories, notwithstanding similar clinical conditions. All of the 4 children we have described present with a comparable clinical history: a prelingual deafness stemming from a genetic aetiology, a very severe degree of preimplant hearing loss (90-100 dB), a good postimplant auditory threshold (25 to 38 dB), and a systematic speech therapy. Yet, the good results of the postimplant pure-tone audiometry were in contrast with the results of the speech audiometry: two of the children fared very poorly, whereas the other two children gained good results. A closer linguistic analysis replicated the findings from the speech audiometry by showing that Participant 1 and Participant 4 were markedly impaired in all the components of the language system that we had examined (lexical, morphological, syntactic, and narrative). The other two children, Participant 2 and Participant 3, obtained scores that fell perfectly within the normal range in all verbal domains. Furthermore, the impairments in the comprehension of language did extend into the corresponding domains of language production, as revealed by the Boston Naming Test, by the sentence production tests (for preposition and pronominal clitics), and by the pictured story test.

However, nonverbal IQ scores were at normal, or slightly above normal, levels in all 4 children.

Hence, we are left with a paradoxical condition: the faculty of language was simultaneously spared and impaired in children affected by the same pathology and with an (almost) identical clinical history.

Owing to this clinical picture, we suspect that, in our cases, linguistic discrepancies of such magnitude and selectivity could hardly be accounted for by purely acoustic variables: indeed, the four children had similar preimplant auditory threshold and an (almost) equivalent postimplant auditory efficiency. Similarly, we could rule out any causal role for the nonverbal IQ, as well as for the educational (same protocols of speech therapy and same rehabilitation programs), socio-economic status, or emotional factors.

Hence, we are left with the possibility that, in certain circumstances, the linguistic brain of some implanted children may not be fully equipped for the computation of language, irrespective of hearing efficiency [33, 34] and for those children the acquisition of language may follow profoundly divergent patterns. Our results are in line with the study of Nittrouer et al., which shows that in spite of early intervention, children with CI are still delayed in learning language and that grammatical knowledge is less affected than phonological awareness [35].

## 6. Conclusion

This line of reasoning brings to the mind some observations made by Fry several decades ago [36] when he wrote that “Again and again children have been found with a comparatively mild degree of hearing loss not exceeding 50 dB in either ear, whose ability to use speech is very poor indeed. On the other hand, there are many instances of children with very considerable losses of hearing of the order of 80-100 dB, who have learned to take in speech through their hearing and have also learned to produce speech that is readily intelligible to the ordinary listeners” (p.148). In an even earlier paper, in 1966, Fry [37] acutely pointed out that “the amount of speech a child develops depends not so much on the amount of hearing per se as upon the use he is able to make of his hearing for language learning” (p.201). Accordingly, we would like to extend Fry's reasoning to the condition of our implanted children by suggesting that the failure of Cases 1 and 4 in acquiring language may not be the sole consequence of a deficient auditory perception, but it may arise from a concomitant impairment of those brain structures involved in language computation, whereas this was not the case for the other two patients.

We are fully aware of the limitations inherent in case series studies [38] and of the risks of attempting to draw general conclusions from the analysis of a few clinical cases. In particular, one of the main limitation of this study is the absence of a neurobiological substrate and of neurofunctional correlates, which are crucial if we aim to explain the correlation between language acquisition delay and biological data. Despite these caveats, our results are sufficiently reliable to suggest that, in CI implanted children, even in the absence of general cognitive deficits and in the presence of a good postimplant pure-tone audiometry, language faculty, in terms of linguistic brain networks and linguistic system development, can be selectively impaired.

Yet, we think that Fry's suggestions are worth deserving a careful reconsideration as they can provide a working hypothesis for a scrutiny of the mechanisms underlying the “variability” of the language outcome in many implanted children and we can conclude that for these children, neither improvements in auditory perception nor an early age of implant per se suffices for a plain development of language.

Mutations in the gene for connexin 26, GJB2, are responsible for a nonsyndromic hearing loss that is usually accompanied by normal vision, vestibular responses, and no malformations at CT scan; varying skin phenotypes including palmoplantar keratoderma or keratitis-ichthyosis-deafness have been described associated with hearing deafness in case of autosomical dominant deafness. No other associated symptoms or signs have been reported before our study [39, 40].

We can make the hypothesis that the neuronal structural damage in children suffering from congenital deafness could in some case diffuse to brain networks involving in language development. For such children, the normalization of auditory threshold could not allow a normal language acquisition because of the impairment of the linguistic system that can be associated with this syndrome.

## Figures and Tables

**Table 1:** Preimplant unaided and aided pure-tone average audiometry. Postimplant pure-tone average and speech audiometry in the four implanted children.

	Participant 1	Participant 2	Participant 3	Participant 4
Unaided pure tone average (across 0.25 to 2.0 khz)	100 dB HL	100 dB HL	100 dB HL	105 dB HL
Hearing aid pure tone average (across 0.25 to 2.0 khz)	63 dB HL	65 dB HL	62 dB HL	71 dB HL
Age at cochlear implant	5.6 yrs	5.5 yrs	4.10 yrs	3.8 yrs
CI pure tone average (from 0.25 to 4.0 khz)	30 dB HL	25 dB HL	38 dB HL	30 dB HL
CI speech audiometry at 50 dB (% of words correctly understood)	40	90	70	25

**Table 2:** Clinical data and IQ scores from the WISC-R scale.

	Participant 1	Participant 2	Participant 3	Participant 4
Chronological age	11.3 yrs	11.4 yrs	9.1 yrs	9.6 yrs
Age at hearing aids	2 yrs	1.8 yrs	1.8 yrs	1.7 yrs
Age at cochlear implant	5.2 yrs	5.5 yrs	4.10 yrs	3.8 yrs
Full scale IQ	73	89	100	80
Performance IQ	100	101	108	115
Verbal IQ	47	80	94	61

**Table 3:** Correct scores (and normative values for chronological age) from the lexical production (Boston Naming Test) and the lexical comprehension (PPVT) tests.

	Participant 1	Participant 2	Participant 3	Participant 4
Chronological age	**11.3** yrs	**11.4** yrs	**9.1** yrs	**9.6** yrs
Boston Naming Test	**19** ^∗∗^ (41.27)	**37** (41.27)	**40** (34.9)	**19** ^∗∗^ (34.9)
PPVT	**59** ^∗∗^ (123)	**124** (125)	**149** (115)	**54** ^∗∗^ (99)

^∗∗^Statistically significant.

**Table 4:** Correct scores from the TROG (Test for Reception of Grammar) and comparison with the normative means for correct scores.

	Total score (items 1-80)	Lexical score (items 1-40)	Grammatical score (items 41-80)	Blocks passed
Participant 1 (11.3 yrs)	**51** ^∗^	**32** ^∗^	**19** ^∗^	**6** ^∗^
	*M* = 77.83	*M* = 39.77	*M* = 38.06	*M* = 18.3
	SD = 2.55	SD = 0.82	SD = 2.39	SD = 1.81

Participant 2 (11.4 yrs)	**76** ns	**40** ns	**37** ns	**18** ns
	*M* = 77.83	*M* = 39.77	*M* = 38.06	*M* = 18.3
	SD = 2.55	SD = 0.82	SD = 2.39	SD = 1.81

Participant 3 (9.10 yrs)	**77** ns	**40** ns	**37** ns	**18** ns
	*M* = 76.54	*M* = 39.7	*M* = 36.84	*M* = 17.51
	SD = 3.47	SD = 0.57	SD = 3.31	SD = 2.25

Participant 4 (9.6 yrs)	**52** ^∗^	**34** ^∗^	**18** ^∗^	**8** ^∗^
	*M* = 76.54	*M* = 39.7	*M* = 36.84	*M* = 17.51
	SD = 3.47	SD = 0.57	SD = 3.31	SD = 2.25

^∗^Statistically significant.

**Table 5:** Error scores from the TCGB (Test of Grammatical Comprehension) for each implanted deaf child.

Errors type	Participant 1 (11.3 yrs)	Participant 2 (11.4 yrs)	Participant 3 (9.10 yrs)	Participant 4 (9.6 yrs)
Locative	**1.5**	**0**	**0**	**3**
Flexional morphology	7.5	0	0	4.5
Active affirmative	6	0	0	3
Active negative	1.5	0	0	1.5
Passive affirmative	7.5	0	0	10.5
Passive negative	4.5	0	0	3
Relative sentences	3	0	0	0.5
Dative sentences	0.5	0	0	0
Total error scores	**32**	**0**	**0**	**26**
Normative error scores for age 8.0 yrs	**1.7**	**1.7**	**1.7**	**1.7**

**Table 6:** Morphological errors (raw and *z* scores) in elicited picture stories (PFLI) in deaf children. Mean correct scores from the control sample.

Pictured scenes (PFLI) (*n* = 10)	Participant 1 (11.3 yrs)	Participant 2 (11.4 yrs)	Participant 3 (9.10 yrs)	Participant 4 (9.6 yrs)	Control sample (*n* = 15) (mean age 7.4 yrs)
Morphemes	**27** ^∗∗^	**0**	**1**	**41** ^∗∗^	**3.2**
Total error score	*z* = 8.92 **(*p* < 0.0001)**	*z* = -1.22 **(*p* < 0.11)**	*z* = -0.85 **(*p* < 0.18)**	*z* = 14.19 **(*p* < 0.0001)**	(2.6)
Free morphemes	**19** ^∗∗^	**0**	**1**	**32** ^∗∗^	**2.8**
Total error score	*z* = 6.52 **(*p* < 0.0001)**	*z* = -1.16 **(*p* < 0.1251)**	*z* = -0.75 **(*p* < 0.1251)**	*z* = 11.77 **(*p* < 0.0001)**	(2.4)
Bound morphemes	**8** ^∗∗^	**0**	**0**	**9** ^∗∗^	**0.4**
Total error score	*z* = 12.02 **(*p* < 0.0001)**	*z* = -0.63 **(*p* < 0.2643)**	*z* = -0.63 **(*p* < 0.2643)**	*z* = 13.60 **(*p* < 0.0001)**	(0.6)

^∗∗^Statistically significant.

## Data Availability

Data are available in the medical database of the Child Neuropsychiatry Unit, Medicine and Surgery Department of the University of Parma.
